# Functions and mechanisms of lactylation in carcinogenesis and immunosuppression

**DOI:** 10.3389/fimmu.2023.1253064

**Published:** 2023-08-14

**Authors:** Jing Su, Zhuangzhuang Zheng, Chenbin Bian, Sitong Chang, Jindian Bao, Huiyuan Yu, Ying Xin, Xin Jiang

**Affiliations:** ^1^ Jilin Provincial Key Laboratory of Radiation Oncology & Therapy, The First Hospital of Jilin University, Changchun, China; ^2^ Department of Radiation Oncology, The First Hospital of Jilin University, Changchun, China; ^3^ NHC Key Laboratory of Radiobiology, School of Public Health of Jilin University, Changchun, China; ^4^ Key Laboratory of Pathobiology, Ministry of Education, Jilin University, Changchun, China

**Keywords:** immunosuppression, lactylation (Kla), metabolic reprogramming, tumor microenvironment (TME), Warburg effect

## Abstract

As critical executors regulating many cellular operations, proteins determine whether living activities can be performed in an orderly and efficient manner. Precursor proteins are inert and must be modified posttranslationally to enable a wide range of protein types and functions. Protein posttranslational modifications (PTMs) are well recognized as being directly associated with carcinogenesis and immune modulation and have emerged as important targets for cancer detection and treatment. Lactylation (Kla), a novel PTM associated with cellular metabolism found in a wide range of cells, interacts with both histone and nonhistone proteins. Unlike other epigenetic changes, Kla has been linked to poor tumor prognosis in all current studies. Histone Kla can affect gene expression in tumors and immunological cells, thereby promoting malignancy and immunosuppression. Nonhistone proteins can also regulate tumor progression and treatment resistance through Kla. In this review, we aimed to summarize the role of Kla in the onset and progression of cancers, metabolic reprogramming, immunosuppression, and intestinal flora regulation to identify new molecular targets for cancer therapy and provide a new direction for combined targeted therapy and immunotherapy.

## 1 Introduction

Metabolic reprogramming is a vital characteristic of tumor cells and plays an important role in tumor therapy, with the Warburg effect being the most widely studied. Even when sufficient oxygen is available to support mitochondrial oxidative phosphorylation, tumor cells tend to convert glucose into lactate (1). Lactate was formerly considered to be a metabolic waste; however, recent research has shown that it can promote the formation and development of cancers through activating the G_i_ protein-coupled receptor 81 (GPR81) signaling pathway (2), influencing cell metabolism (3), and modulating the tumor microenvironment (TME) (4).

Protein posttranslational modifications (PTMs) refer to the process of changing the biochemical properties of proteins through adding chemical groups to one or more amino acid residues so that precursor proteins can have specific functions. Common modifications include acetylation, ubiquitination, methylation, phosphorylation, and glycosylation (5). PTMs are closely related to carcinogenesis, and their pathological significance involves all cancer characteristics, including maintenance of proliferation signals, resistance to cell death, induction of angiogenesis, and activation of invasion (6). PTMs also play vital roles in TME regulation. Swamy et al. (7) found that activated effector T cells contain more O-GlcNAcylation-modified proteins than naïve T cells, suggesting that O-GlcNAcylation plays an important role in T cell activation. Forkhead/winged helix transcriptional factor P3 (FoxP3) is a transcriptional regulator that plays an important role in regulatory T cell (Treg) growth. Acetylation can enhance its ability to bind to chromatin, thereby increasing the number and activity of Treg cells and inhibiting the antitumor effects of the immune system (8). Therefore, targeting PTMs to key proteins or pathways is an emerging strategy for improving early cancer detection and treatment.

Lactylation (Kla) is a novel PTM. Zhao et al. (9) conducted the first study on histone Kla in 2019, demonstrating that Kla refers to the addition of a lactyl (La) group to a lysine residue in the histone tail. Later studies established that Kla is prevalent in immune-related cells (10), non-small cell lung cancer (NSCLC) (11), and ocular melanoma (12), and that it is strongly associated with the development of malignancies (13). This systematic review aimed to examine the important role of Kla in tumor cell metabolism, microenvironment, and immunosuppression, explore the possibility of targeting Kla sites and catalytic enzymes for cancer therapy, and provide a new direction for combined targeted cancer therapy and immunotherapy.

## 2 Lactate regulation of tumor metabolism and microenvironment

**Figure 1:**
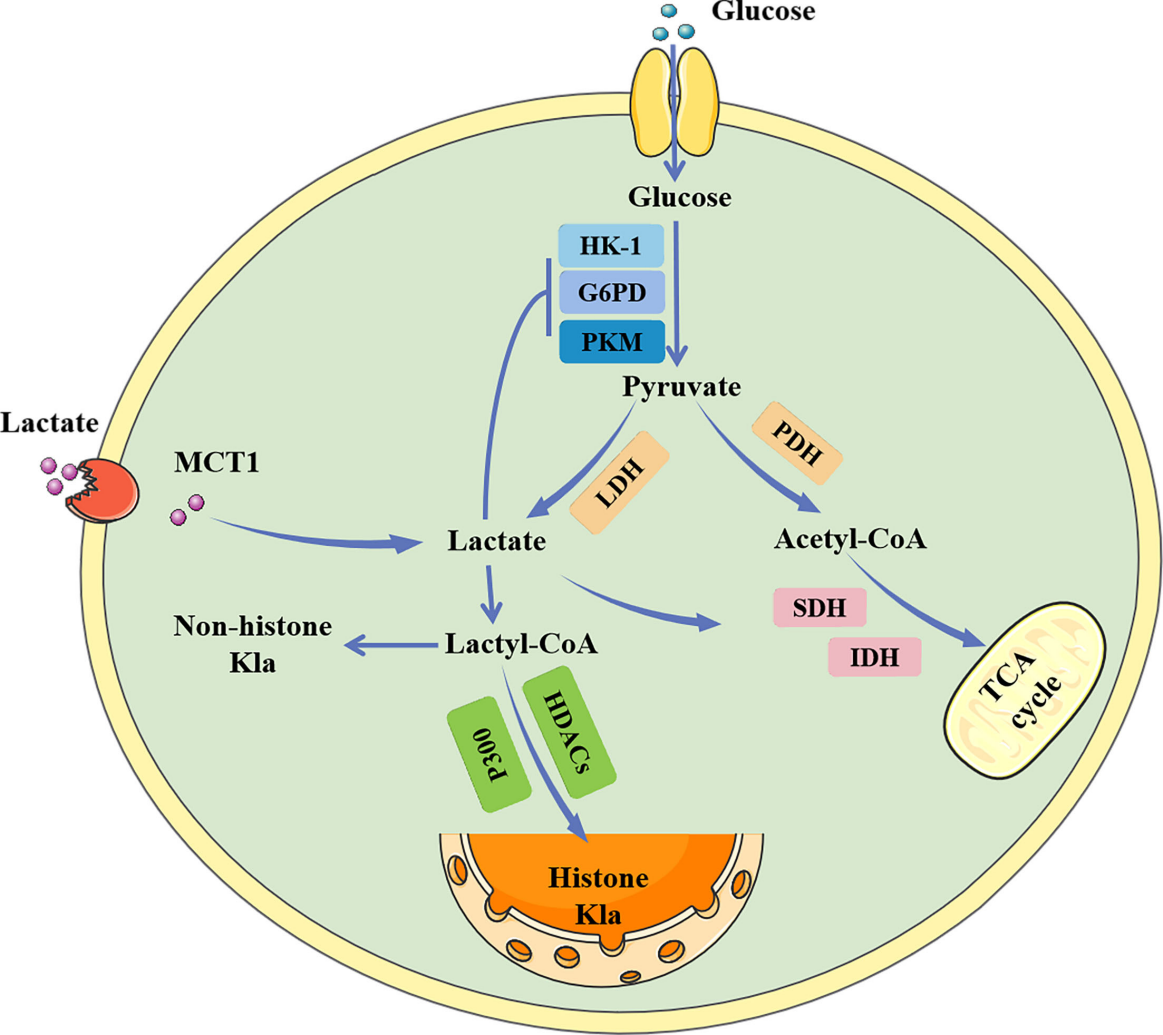
The metabolic process of lactate. Lactate can be created intracellularly by glycolysis catalyzed by LDH, or it can be taken up from the outside via MCT1. Intracellular lactate inhibits glycolytic enzymes while promoting the tricarboxylic acid cycle, generating a negative feedback loop. Lactate can also be converted to lactoyl-CoA, which is involved in the lactylation of histone and non-histone proteins. HK-1, hemokinin-1; G6PD, glucose 6-phosphate dehydrogenase; PKM, glycolytic enzyme pyruvate kinase M; MCT1, monocarboxylate transporters 1; LDH, lactate dehydrogenase; PDH, Pyruvate dehydrogenase; SDH, succinate dehydrogenase; IDH, isocitrate dehydrogenase; TCA, Tricarboxylic acid; HDAC, histone deacetylase; Kla, lactylation.

Glucose primarily generates energy *via* two metabolic pathways: glycolysis and oxidative phosphorylation (**Figure 1**). Both pathways begin with the conversion of glucose to pyruvate, followed by the production of adenosine triphosphate (ATP) and nicotinamide adenine dinucleotide (NADH). When oxygen is available, the pyruvate produced during glycolysis is carried into the mitochondria and transformed into acetyl-CoA, which subsequently enters the tricarboxylic acid (TCA) cycle and generates considerable energy (14, 15). However, in the absence of oxygen, pyruvate is converted to lactate by lactate dehydrogenase (LDH) (16). Under aerobic conditions, the glycolytic process in normal mammalian cells is blocked, which is known as the Pasteur effect (17). However, the glycolytic metabolism of tumor cells is significantly active even in the presence of an ample oxygen supply, which is known as the Warburg effect (1).

The causes of the Warburg effect remain contentious, and several theories provide preliminary explanations. One possible explanation is that, under oxygen-sufficient conditions, the efficiency of ATP production is not a limiting factor for cell multiplication. When the capability of glucose metabolism to produce ATP is impaired in normally proliferating cells, they undergo cell cycle arrest and reactivate catabolism while activating signaling pathways, such as AMP-activated protein kinase (AMPK) and activating protein kinase B (AKT), to maintain energy homeostasis (18, 19). Furthermore, cancer cells constantly divide and require additional metabolic intermediates to form macromolecules for their daughter cells. However, mitochondrial oxidative phosphorylation converts all glucose into CO_2_ to maximize ATP generation, in conflict with the requirements of growing cells. During glycolysis, glucose can be transformed into additional macromolecular precursors, such as acetyl-CoA for fatty acid synthesis, glycolytic intermediates for nonessential amino acid synthesis, and ribose for nucleotide synthesis, to enhance cell proliferation (9). In addition, it has been shown that adverse metabolic conditions in TME can lead to the activation of transcription factors such as Krüppel-like factor 4 (KLF4) and nuclear factor-kappa B (NF-κB), leading to the selection of the Warburg phenotype through transcriptional reprogramming (20).

Previously, the lactate produced through glycolysis was considered to be merely metabolic waste. However, in recent years, an increasing number of studies have shown that lactate plays a role in tumor growth. Lactate can be employed as a GPR81 ligand to activate the GPR81 signaling pathway, which can then influence the expression of metabolism-related genes and promote tumor growth (2). In addition, Vegran et al. (21) found that lactate can enter endothelial cells through monocarboxylate transporter 1 (MCT1), causing degradation and phosphorylation of IκBα, and then stimulating the autocrine NF-κB/interleukin (IL)-8 pathway to cause cell migration and blood vessel formation. Lactate is also closely related to TME (22). Proton-coupled lactate transport in cancer or stromal cells creates an acidic environment with a pH ranging from 6–6.6, leading to tumor development and treatment resistance (23). Unlike exogenous pathogens, cancer cells are derived from normal cells and express almost all the proteins that normal cells express, so they are more difficult to distinguish by the immune system. In lymphocytes, B cells can act as antigen-presenting cells and secrete cytokines to exert anti-tumor effects (24); Cytotoxic T lymphocytes (CTLs) can specifically recognize tumor-associated antigens through major histocompatibility complex (MHC) I on their surface, bind to tumor cells and produce perforin and other cytotoxins to kill cancer cells (25); Natural killer (NK) cells have no MHC or human leukocyte antigen restriction and can release perforin, granzyme and cytokines to destroy tumor cells (26). Among myeloid cells, the frequency of dendritic cells in tumors was associated with a good prognosis, while tumor-associated macrophages (TAM), especially M2 macrophages, were associated with a poor prognosis (27). It has been reported that lactate generated from tumor cells can activate the production of vascular endothelial growth factor (VEGF) and arginase 1 (Arg1), promoting TAM polarization to M2 and assisting TAM in promoting tumor growth (28). Besides, the lactate concentration in the TME can limit the lactate efflux of CTLs, lowering the production of cytokines, perforin, and granzyme B, and inhibiting CTL cytotoxicity (29). NK cells are potent innate immune effectors that serve as the first line of defense against cancer. Lactate induces NK cell apoptosis (30). Natural killer T (NKT) cells are another type of immune cells with anti-tumor activity. Lactate can inhibit the production of interferon-γ (IFNγ) and IL-4 by NKT cells, inhibit their survival and proliferation, and promote tumor development (31).

However, the specific mechanisms underlying lactate uptake and utilization by tumor cells have not yet been fully elucidated. The accumulation of lactate in the TME can be reduced through increasing its uptake and utilization by tumor cells, thus reducing its influence on tumor cells and immune cells, or through finding effective targets to cause tumors to undergo aerobic metabolism instead of the Warburg effect, thereby inhibiting tumor invasion. These issues require urgent attention in the field of lactate metabolism in tumors.

## 3 Lactylation: a new posttranslational modification

Kla is a PTM protein first reported in 2019 (32). Subsequent studies have further confirmed that Kla has an important function in relation to lactate and is involved in important activities such as tumor proliferation (33), nervous system regulation (34), and metabolic regulation (35).

Lysine is a basic amino acid and its sixth amino group is highly active. After modification, this amino acid changes from basic to acidic, leading to changes in protein polarity and function (36). Lysine acylation is a widespread and highly conserved PTM that affects various physiological and pathological processes through epigenetic regulation. Using mass spectrometry analysis, Zhao et al. (32) found a mass shift of 72.021 Da on lysine residues in three proteolytic peptides, which increased in a dose-dependent manner with an increase in lactate. Subsequently, they showed the existence of Kla through tracking isotopic sodium l-lactate (^13^C_3_). Inhibition of pyruvate dehydrogenase kinase (PDK) and LDH production by dichloroacetic acid (DCA) and oxalate was found to cause Kla inhibition (37, 38), whereas inhibition of mitochondrial oxidative phosphorylation by rotenone caused an increase in Kla. This implies that lactate levels are inextricably linked to local lactate concentrations. Furthermore, stressors such as hypoxia and bacterial infection can promote a change in cellular energy metabolism into a glycolysis-dependent pattern, resulting in increased lactate production and the activation of histone Kla (39).

Kla has also been shown to cause changes in the expression of genes related to inflammatory responses (40), macrophage polarization (32), and other processes. Several studies have shown that Kla plays an important role in disease occurrence and development (33, 34). However, research into “writers” and “erasers,” which are necessary for the occurrence and removal of Kla, remains in its infancy and has yet to be expanded.

**Figure 2:**
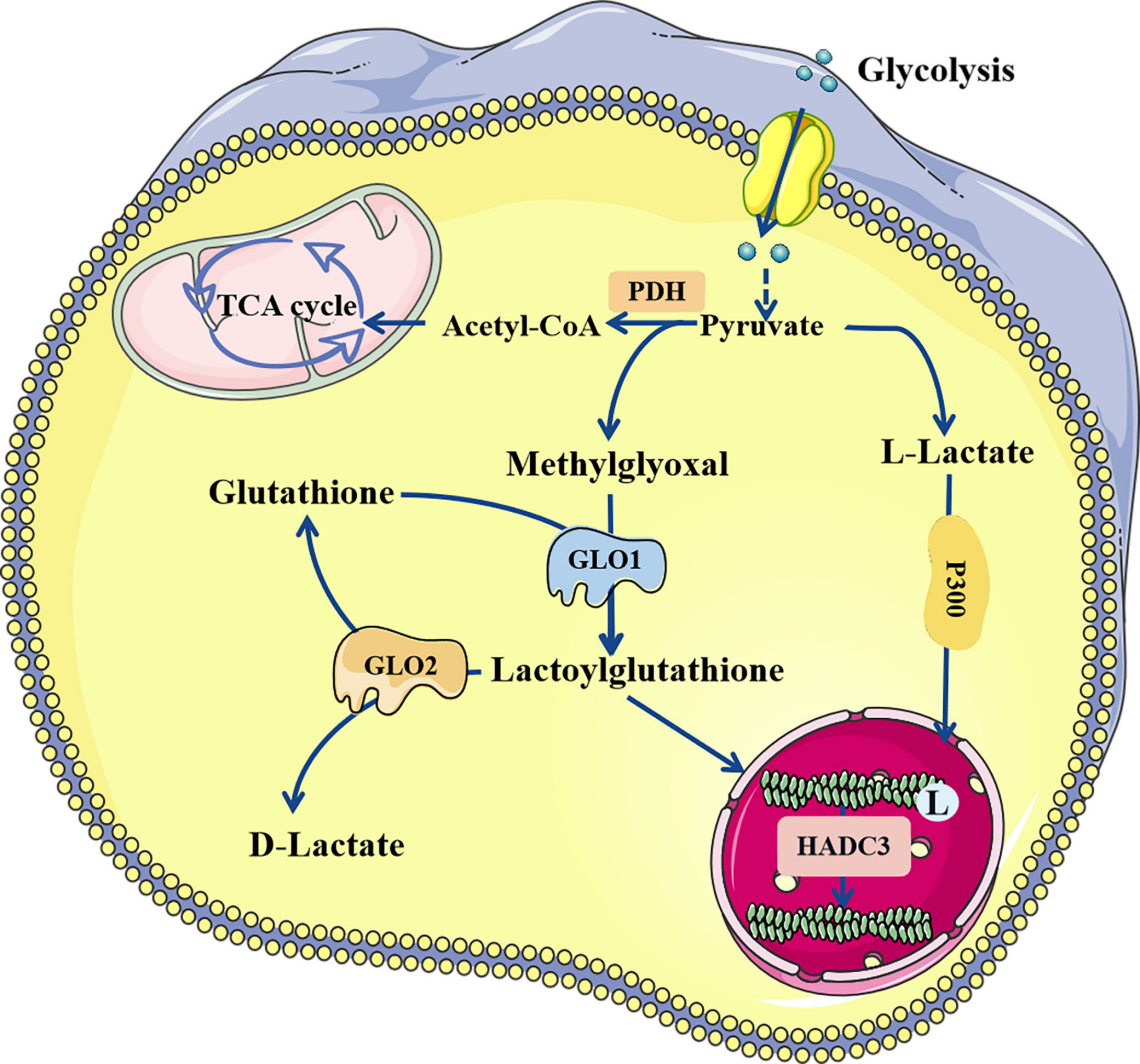
The regulatory process of lactylation. There are two known modification pathways for lactylation. L-lactate can form lactoyl-CoA, which mediates lactylation under the catalysis of P300. In addition, methylglyoxal can form LGSH under the catalysis of GLO1, and LGSH can also mediate the lactylation of proteins, which does not require enzyme catalysis. LGSH can be decomposed into glutathione and D-lactate under the action of GLO2. Both L-and D-lactate-mediated lactylation were abolished by HDAC3. LGSH, Lactoylglutathione; GLO, glyxoalase.

Lactate, a chiral molecule, typically exists as three optical isomers: D-lactate, L-lactate, and racemic DL-lactate. L-Lactate is the most common type of lactate. It increases dramatically in pathological conditions such as tumors, sepsis, and autoimmune disorders (41). According to previous research, L-lactate can be activated through acyl-CoA synthetase and transported to lysine residues by histone acetyltransferases such as p300 (32). D-Lactate is primarily synthesized in cells through the metabolism of-carbonyl aldehydes with high glycation activity, such as methylglyoxal (MGO) (42). D-Lactate concentrations can increase dramatically when intestinal function is impaired (43). Galligan et al. (44) found that methylglyoxal, a byproduct of glycolysis, can combine with glutathione to form lactoylglutathione (LGSH), which is catalyzed by glyoxalase 1 (GLO1). Simultaneously, GLO2 hydrolyzes LGSH, cycling glutathione, and produces D-lactate. LGSH can be employed as a donor to supply lactate molecules to enhance the Kla of target proteins; this reaction does not require enzyme catalysis. In addition, Zhao et al. (45) found that common deacetylases HDAC1-3 and SIRT1-3 have the ability to eliminate Kla, among which HDAC3 is the most effective “eraser” for L- and D-lactylation (46) (**Figure 2**).

The study of Kla and its associated enzymes offers new perspectives for targeted cancer treatment and has promising practical applications. As an increasing number of histone Kla sites have been identified, the role of Kla in nonhistone proteins is becoming increasingly understood and recognized. Among glycolytic enzymes, Kla has been found to be widespread. For instance, the Kla of aldolase A (ALDOA) is present in numerous human tumor cell lines and can control glycolysis through controlling the activity of metabolic enzymes (47). Through thoroughly analyzing the tumor and nearby liver, Young et al. (48) identified 9,275 Kla sites, of which 9,256 were found on proteins other than histones, indicating that Kla is a broad alteration that extends beyond histone and transcriptional regulation. They also showed that the p53 pathway is controlled by adenylate kinase 2 (AK2) Kla in hepatocellular carcinoma (HCC), which contributes to a poor prognosis.

## 4 Lactylation promoting the occurrence and development of tumors

### 4.1 Lactylation affecting the metabolism of tumor cells

The metabolic reprogramming of tumor cells, which promotes rapid cell growth and proliferation through altering the metabolism, is considered to represent a new cancer characteristic (49). The most prevalent and basic type of research on tumor metabolism is aberrant glucose metabolism (50). Tumor cells have a distinctive glucose metabolism pattern compared with normal cells; even under oxygen-sufficient conditions, they prefer glycolysis to the tricarboxylic acid (TCA) cycle to obtain energy. Recent studies have also found that many other metabolic pathways, including fatty acid metabolism (51), cholesterol metabolism (52), and glutamine metabolism (53), undergo reprogramming changes in tumor cells.

Hypoxia-inducible factor alpha (HIF-1α) is a key gene that regulates the metabolic mode of tumor cells (54). Lactate concentration can inhibit the activity of proline hydroxylase (PHD) and weaken the ubiquitination and degradation of HIF-1α by PHD, which indicates that lactate is closely related to tumor metabolism (55). Previous studies have demonstrated that lactate can be used as a source of three-carbon compounds in mammals. Circulating lactate also allows uncoupling of glycolysis and TCA, allowing glucose use to be regulated according to more advanced bodily demands (56). Besides, lactate and pyruvate together can be used as a circulating REDOX buffer to balance the NADH/NAD ratio in cells and tissues (57). Chiarugi et al. (3) demonstrated that lactate can activate the key enzymes ATP citrate lyase and acetyl-CoA carboxylase in fatty acid synthesis, as well as the cholesterol pathway, by redirecting citrate in prostate cancer cells. Meanwhile, the current study showed that the accumulation of circulating lactate is negatively correlated with fat oxidation (58). In recent years, research concerning Kla has gain considerable momentum, and its role in tumor metabolism has gradually been revealed.

Wan et al. (47) found that Kla regulates numerous glycolysis-related enzymes. For example, Kla at the K147 site of fructose-bisphosphate ALDOA is found in both humans and animals. After Kla treatment, ALDOA activity was reduced, and its catalytic function was suppressed, establishing a negative feedback loop. Kla may also influence the function of other enzymes involved in glycolysis. Kla of the human recombinant enolase (ENO1) disrupts its interaction with substrates. Given the prevalence of metabolic dysfunction in the TME and high lactate levels resulting from the Warburg effect, it is plausible that Kla serves as a crucial link between lactate, tumor metabolism, and patient prognosis.

In a study concerning hepatocellular cancer, Kla was found to have primarily affected enzymes involved in metabolic pathways such as the TCA cycle, glucose, amino acid, fatty acid, and nucleotide metabolism. Kla at the K28 site then inhibited the function of AK2 and promoted the growth and spread of liver cancer cells (48). Studies concerning NSCLC have shown that increased histone Kla levels may cause downregulation of hemokinin-1 (HK-1) and pyruvate kinase M (PKM) in glycolysis, and upregulation of succinate dehydrogenase (SDHA) and isocitrate dehydrogenase 3 gamma (IDH3G) in the TCA cycle. As a result, glucose absorption and glycolysis are inhibited, mitochondrial homeostasis is maintained, and tumor development occurs (11). Furthermore, Kla has been shown to control the activity of enzymes involved in lipid metabolism in a nonalcoholic fatty liver disease model. The presence of Kla at the K673 site of fatty acid synthase inhibits its function and decreases lipid accumulation in the liver (59).

Tumor metabolic reprogramming can successfully increase tumor cell proliferation, growth, migration, and invasion, among other important biological functions (49). Therefore, targeting tumor metabolism through exploiting metabolic variations between tumor and normal cells is a promising anticancer technique. As a new PTM, Kla plays an important role in the regulation of tumor metabolism, especially glucose metabolism. However, there have been few investigations of other basic metabolic pathways such as lipid and amino acid metabolism. Further exploration of the regulatory mechanism of Kla in tumor metabolism and the search for more Kla sites could provide more reliable targets for tumor therapy.

### 4.2 Regulation of TME

Changes in TME are also important factors affecting carcinogenesis. Lactate is one of the most prominent metabolites in TME. Lactate produced by tumor cells via the Warburg effect can enter the extracellular microenvironment and promote angiogenesis through upregulating the expression of angiogenesis-related proteins. At the same time, it can regulate the metabolism of immune cells and inhibit the proliferation of CD8^+^ T cells, NK cells, and dendritic cells, thereby mediating immune escape (60, 61). The degree of histone lysine Kla has been found to be higher in TAM than in other tissues, implying that Kla plays an important regulatory role in TME, which may provide a new direction for tumor immunotherapy, antiangiogenesis therapy, and targeted therapy.

Lactate accumulation can stimulate angiogenesis by activating VEGF, transforming growth factor (TGF), IL-1, HIF-1α, and other substances (62, 63). According to previous studies, Kla plays a crucial role in this process. The high expression of hyaluronic acid (HA)-binding protein KIAA1199 has been reported to be positively correlated with tumor stage, overexpression of HIF-1α and upregulation of angiogenesis markers (64, 65). Lactate enters prostate cancer cells via MCT1 and promotes the Kla of HIF-1α, keeping it stable under normoxic circumstances and boosting KIAA1199 transcription. This discovery could lead to a promising target for antiangiogenic tumor therapy (66).

**Figure 3:**
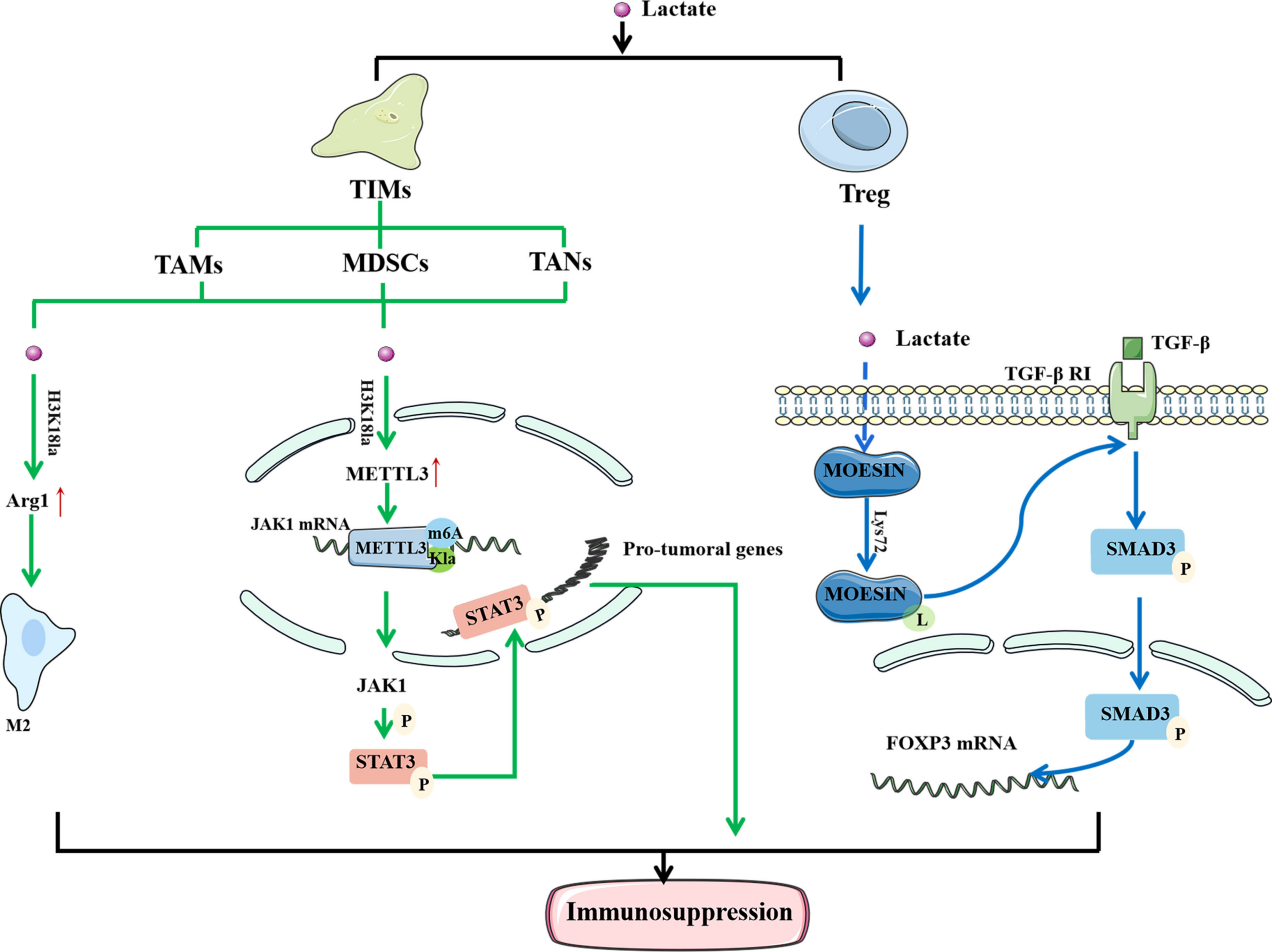
Lactylation regulates the function of immune cells to mediate immunosuppression. In TAM, histone lactylation will lead to the up-regulation of M2 phenotypically related genes, thereby mediating the M2 polarization. In TIMs, histone lactylation promote the expression of METTL3, and METTL3 mediates the m6A modification of JAK1 mRNA. At the same time, there are also lactylation sites in METTL3 that allow it to bind to the target RNA. Moesin can reduce the expression of TGF-β receptor and inhibit the production of Treg cells to restore anti-tumor immunity. Lactate can inhibit the function of moesin by promoting Lys72 site lactylation to mediate the generation of Treg cells and promote the immune escape of tumor cells. In conclusion, lactylation is closely related to the immunosuppression of tumors. LPS, Lipopolysaccharide; IFN γ, Interferon-gamma; IL-4, interleukin-4; IL-13, interleukin-13; TAM, tumor-associated macrophages; TIM, tumor -infiltrating myeloid cells; Arg1, arginase 1; METTL3, methyltransferase-like 3; JAK1, Janus Kinase 1; M6A, N6-methyladenosine; TGF-β, transforming growth factor beta.

Immune cells play a significant anticancer role in TME. They can detect extracellular lactate levels and send intracellular signals to alter their function in TME (29). As a result, the impact of Kla on the efficacy of immunotherapy has attracted interest (**Figure 3**). Tumor-infiltrating myeloid cells (TIMs) include TAMs, myeloid-derived suppressor cells (MDSCs), and tumor-associated neutrophils (TANs). They are closely related to tumor immune escape and their functions are regulated through a variety of epigenetic processes. The increased expression of methyltransferase-like 3 (METTL3) in TIMs has been linked to poor prognosis in patients with colon cancer (67). Lactate stimulates METTL3 expression in TIMs through the H3K18 Kla. METTL3 mediates the m6A modification of Janus kinase (JAK1) mRNA, enhancing its protein translation efficiency and mediating an increase in the phosphorylation level of its downstream protein signal transducer and activator of transcription 3 (STAT3). Phosphorylated STAT3 can act as a transcription factor to regulate the production and secretion of cytokines, such as IL-6 and IL-10, resulting in immunosuppression. In addition, the presence of two Kla sites in the zinc-finger domain of METTL3, which are required for it to capture target RNA, has been confirmed. These findings highlight the importance of Kla in promoting the immunosuppressive capacity of TIMs (68).

Macrophages are one of the most important innate immune cells in the human body and are classified into M1 and M2 phenotypes. Lipopolysaccharide (LPS) and IFNγ activate M1 macrophages, while IL-4 or IL-13 stimulate M2 macrophages (69). TAMs are common in the TME and associated with the formation and progression of malignancies (70). Seventy percent of TAMs exhibit the M2 phenotype, which suppresses the immune system and promotes tumor development and metastasis (71). Studies have shown that M1 macrophages undergo metabolic reprogramming during aerobic glycolysis to produce lactate during polarization. In contrast, polarization of M2 macrophages enhances aerobic phosphorylation and fatty acid metabolism (72). Zhao et al. (32) found that activation of M1 macrophages leads to the activation of glycolysis, accompanied by the production of a large amount of lactate and upregulation of H3K18la. Histone Kla can cause an increase in M2 phenotype-related proteins, indicating that it promotes the transformation of TAMs from the proinflammatory, anticancer M1 phenotype to the antiinflammatory, procancer M2 phenotype. In a study concerning prostate cancer, Patnaik et al. (73) discovered that reducing phosphatidylinositol-3 kinase (PI3K) resulted in a decrease in the synthesis of lactate in tumor cells, thereby inhibiting the histone Kla of TAM and enhancing immune efficacy. In conclusion, histone Kla is closely related to M2 polarization of TAMs, and increased levels of Kla tend to cause immunosuppression and reduce the efficacy of immunotherapy.

TGF-β is a multifunctional cytokine belonging to the transforming growth factor superfamily. It plays a crucial role in stem cell differentiation and T cell regulation (74). When activated, T cells effectively attack and kill tumor cells. However, when the immune response is complete, TGF-β delivers a signal to naïve T cells to become Treg cells to regulate and destroy activated proinflammatory T cells, ensuring that they do not create too many immune factors and cause damage to their own cells and tissues (75). In tumor tissues, Treg cells suppress immune responses and protect cancer cells from killer T cells. Moesin reduces the expression of TGF-β receptors and inhibits the production of Treg cells to restore antitumor immunity (76). Studies have found that lactate can inhibit the function of moesin by promoting Kla at the Lys72 site to mediate the generation of Treg cells and promote the immune escape of tumor cells. Therefore, it is feasible to improve the efficacy of immunotherapy by inhibiting moesin Kla (10). In summary, the discovery of Kla provides a new direction for tumor immunotherapy.

### 4.3 Lactylation affecting the growth and distribution of intestinal flora

Kla provides a new perspective on the regulatory function of lactate, which plays an important role in eukaryotic cell metabolism and gene transcription (32). Lactate is a key carbon source for bacteria and is linked to stress tolerance, cell wall remodeling, and virulence (77, 78). However, if Kla occurs in prokaryotes, the biological functions it may be involved in need further investigation.

YiaC and CobB are common lysine acetyltransferases and deacetylases found in prokaryotes, respectively (79). Dong et al. found that YiaC catalyzes Kla in *Escherichia coli*, whereas CobB scavenges PTM. CobB enhances glycolysis and bacterial growth through eliminating the Kla of pyruvate kinase I (PykF) (80), which provides a strong basis for controlling the growth and distribution of certain bacteria through regulating specific Kla sites.

**Figure 4:**
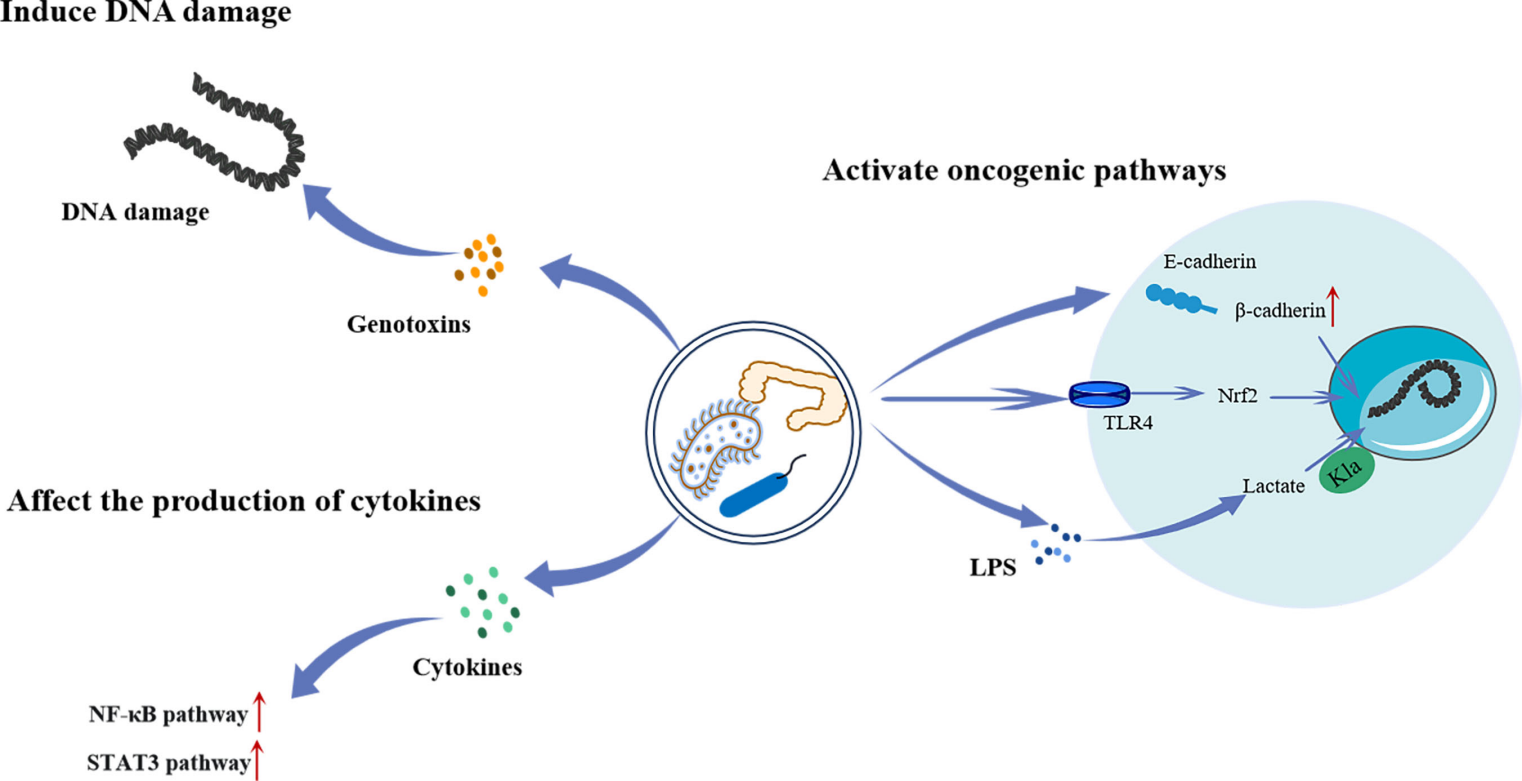
The mechanism of intestinal flora regulating the occurrence and development of tumors. Intestinal bacteria can directly cause intracellular DNA damage by producing genotoxins, or activate the NF-κB and STAT3 pathway by promoting cytokines secretion to promote tumorigenesis. Intestinal bacteria can also activate a range of oncogenic pathways. Increased release and translocation of β-catenin into the nucleus through degradation of the E-cadherin/β-catenin complex leads to aberrant activation of WNT signaling associated with various cancers. Besides, they can lead to the invasion and metastasis of cancer by activating TLR4/Keap1/NRF2 signaling. Furthermore, LPS produced by bacteria has been shown to promote the Kla of histones, which in turn promotes tumor progression. LPS, Lipopolysaccharide; TLR4, Toll-like receptor 4; Nrf2, Nuclear factor E2-related factor 2; NF-κB, Noncanonical nuclear factor-kappaB; STAT3, ignal transducer and activator of transcription 3.

Intestinal flora refers to a group of bacteria that live in the intestinal tract of humans and animals. They play important roles in regulating digestion, immunity, and nutrient absorption. In recent years, an increasing number of investigations have shown that certain bacteria can cause malignancies in gastrointestinal tissues (**Figure 4**). For example, *Helicobacter pylori* (HP) is closely related to atypical hyperplasia, intestinal metaplasia, and gastric cancer progression (81). Moreover, *Fusobacterium nucleatum* is closely related to the transformation of colorectal adenomas into adenocarcinomas (82). The carcinogenic mechanism of the intestinal flora is not yet fully understood. Several studies have shown that some members of *Enterobacteriaceae* produce colibactin, which results in DNA damage and promotes malignancies (83). In addition, they can affect cytokine synthesis and secretion, thereby activating various carcinogenic pathways (84). Furthermore, several Toll-like receptors (TLR), such as TLR4 and TLR5, have been linked to the interaction between malignancies and microorganisms. Kong et al. (85) found that *F. nucleatum* promotes colorectal cancer by activating TLR4/Keap1/NRF2 signaling. Moreover, studies have shown that lactate regulates the proliferation of intestinal flora and has a carcinogenic effect (86).

Panagiotou et al. (87) found that the excessive growth of intestinal *Candida* in patients with lung cancer was related to an increase in lactic-producing bacteria and a decrease in short-chain fatty acid production. *Candida* species use lactate as a nutrient source to compete with other fungi in the intestine. In addition, gut microbes can participate in disease pathogenesis via their interaction with the host genome through epigenetic mechanisms, such as long non-coding RNA. Liu et al. (88) quantitatively analyzed transcriptomic changes in a human colon cell line after infection with the common intestinal pathogen *Salmonella typhimurium* SL1344. They found that LINC00152 expression was significantly increased and associated with intestinal microbiome-derived LPS. LPS induces histone Kla at the LINC00152 promoter and decreases its binding efficiency to the transcription factor yin-yang 1 (YY1), leading to increased LINC00152 expression, thereby promoting the migration and invasion of colon cancer cells.

Bacteria promote carcinogenesis by causing DNA damage and activating oncogenic pathways. Additionally, bacteria can affect tumors through controlling the body’s immune system and metabolism (89). It has been shown that the level of Kla affects the growth and dissemination of bacteria. However, whether specific Kla sites exist in different bacteria and whether tumor progression can be controlled by modulating the Kla level in the intestinal flora needs to be further explored.

## 5 Lactylation providing a new direction for targeted therapy and immunotherapy

Kla is involved in a variety of processes such as tumor metabolism, angiogenesis, and immunosuppression, and is closely related to the poor prognosis of tumors (32, 47). Therefore, targeting specific sites that inhibit the occurrence of Kla may be an effective cancer treatment strategy (**Table 1**).

**Table 1:** The regulation of lactylation in cancer cells.

Cancers	Lactylated protein(s)/site(s)	Function	References
Prostate cancer	HIF-1α	Promote angiogenesis	(66)
	H3K18	Neuroendocrine differentiation	(90)
T-cell acute lymphoblastic leukemia	ALDOA	Metabolic Reprogramming	(47)
Colorectal cancer	ALDOA	Metabolic Reprogramming	(47)
Liver cancer	AK2	Regulate p53 pathway and lead to poor prognosis	(48)
	CCNE2	Promote tumor proliferation	(91)
	H3K9la, H3K56la	Promote tumorigenesis	(92)
Non-small cell lung cancer	H3	Inhibit glycolysis	(11)
	H3K18	Neuroendocrine differentiation	(90)
Ocular melanoma	H3K18	Upregulate the oncogene YTHDF2 and promote tumorigenesis	(12)
Anaplastic thyroid cancer	H4K12	Cause cell cycle dysregulation and promote tumor proliferation	(93)
Clear Cell Renal Cell Carcinoma	H3K18	Activate the transcription of PDGFRβ and promote tumor proliferation	(94)
Breast cancer	H3K9,18,23,27,56,122 H4K5,8,12,31,77,91	N/A	(9)
Cervical cancer	H3K9,18,23,27,56,122 H4K5,8,12,31,77,91 H2AK11,13,115 H2BK5,11,15,16,20,23,43,85, 108,116,120	N/A	(9)

HIF-1α, Hypoxia-inducible factor-1alpha; ALDOA, aldolase A; AK2, adenylate kinase 2; CCNE2, cyclin E2; YTHDF2, Human YTH domain family 2; PDGFRβ, platelet-derived growth factor receptor β.

N/A means "not applicable". It means that the function of these Kla sites has not been reported.

### 5.1 The role of lactylation in tumor targeted therapy

In tumor cells, glucose undergoes glycolysis to produce pyruvate, which then reacts with LDH to form lactate. Lactate can inhibit glycolysis and promote the TCA cycle by regulating key enzymes and producing a negative feedback loop (95). Studies have shown that Kla levels are positively correlated with intracellular lactate content (32). Therefore, key enzymes involved in lactate metabolism may be potential targets for tumor treatment. LDH is a key enzyme in the conversion of pyruvate to lactate during glycolysis. It maintains glycolysis and ATP production by regenerating NAD to form NADH (96). Baumann et al. (97) showed that the carcinogenicity of LDH-deficient tumor cells was significantly reduced and, after LDH knockdown, the metabolic phenotype of the cells was disturbed and their proliferation ability was significantly decreased. Furthermore, the enzyme ULK1, as an upstream regulator, could mediate the phosphorylation of LDH and enhance its enzymatic activity to promote lactate production, thereby promoting tumor progression (98). In addition to being produced within cells, lactate can be secreted between cells through MCTs to participate in the regulation of cellular physiological and pathological processes (99). Several studies have shown that targeting MCT1 or MCT4 can effectively inhibit the growth of multiple types of tumors, such as breast (100), liver (101), and bladder cancers (102).

p300 and SIRT3 are present as “writer” and “eraser” for Kla. Studies have shown that p300 is involved in tumor proliferation, migration, and invasion (103). SIRT3 can eliminate lysine Kla, and cyclin E2 (CCNE2) acts as a substrate. SIRT3 induces apoptosis in HCC cells through regulating the Kla level of CCNE2 and prevents HCC growth *in vivo* (91). Therefore, further exploration of the mechanism of action of Kla and identification of Kla-related catalytic enzymes may provide more targets for tumor treatment.

In addition to the enzymes related to lactate metabolism and Kla, the identification of an increasing number of Kla and their regulatory sites opens up new directions for tumor therapy. The BRAFV600E oncogene has been shown to increase the level of the intracellular protein Kla through enhancing glycolytic flux. It can promote the proliferation of anaplastic thyroid cancer through inducing H4K12 Kla, mediating driver gene transcription, and cell cycle dysregulation (93). The ninth member of the proprotein convertase family, subtilisin-kexin type 9 (PCSK9), maintains lipoprotein homeostasis. Alterations in PCSK9 expression have been linked to carcinogenesis and progression (104). The levels of lactate, protein Kla, and macrophage migration inhibitory factor (MIF) in colon cancer cells were found to have significantly increased after PCSK9 overexpression, which inhibited the polarization of M1 macrophages and promoted the occurrence and development of colon cancer, providing a potential therapeutic method for the clinical control of colon cancer (33). Pan et al. (92) identified a triterpene antitumor drug that inhibited the carcinogenesis of liver cancer stem cells through interfering with H3K9la and H3K56la Kla. Inactive von Hippel-Lindau (VHL) protein is associated with metabolic reprogramming and is important in the development of clear cell renal cell carcinoma (ccRCC) (105). VHL inactivation promotes the progression of ccRCC through triggering histone Kla to activate the transcription of platelet-derived growth factor receptor β (PDGFRβ). Meanwhile, PDGFRβ signaling in turn stimulates histone Kla, thereby forming a positive feedback loop for carcinogenesis in ccRCC. The combined reduction in histone Kla and PDGFRβ signaling was shown to substantially increase the therapeutic efficacy. This suggests that addressing the positive feedback loop between histone Kla and PDGFRβ signaling could be a viable treatment for patients with ccRCC (94). Additionally, histone Kla could stimulate the production of YT521-B homology domain family member 2 (YTHDF2), which leads to the degradation of m6A-modified period1 and TP53 and accelerates the development of ocular melanoma (12).

It has also been reported that Kla has a role in the neuroendocrine transition of adenocarcinoma (90). Neuroendocrine differentiation in adenocarcinoma is an important cause of resistance to tumor treatment (106). Most neuroendocrine cancer cells are glycolytic in nature and contain fragmented mitochondria with low membrane potential (107). Numb plays a crucial role in mitochondrial quality control by attaching to Parkin and facilitating Parkin-mediated mitophagy (90). In prostate and lung adenocarcinomas, loss of the Numb/Parkin pathway results in metabolic reprogramming marked by a significant increase in lactate generation, which in turn causes an increase in histone Kla and the activation of the transcription of genes linked to neuroendocrine function. Collectively, Numb/Parkin is a promising therapeutic target for modulating cancer cell plasticity by regulating histone Kla.

At present, some drugs targeting lactate metabolism have entered clinical trials. Lonidamine (LND), a dechlorinated derivative of indolazole-3-carboxylic acid, can destroy tumor cells by decreasing lactate production and pyruvate uptake in mitochondria and interrupting the mitochondrial transmission chain. LND does not have good anticancer activity when used alone, but it has been widely researched in combination with standard chemotherapy for the treatment of solid tumors (108). Madrid et al. (109) revealed that the combination of LND and cisplatin was more effective than cisplatin alone in reducing tumor growth in MX-1 breast cancer and A2780 ovarian cancer. Dichloroacetate (DCA) is an oral small-molecule medication that reduces tumor growth by inhibiting pyruvate dehydrogenase kinase and encouraging glucose oxidation rather than glycolysis (110). DCA has shown promising efficacy in both recurrent glioblastoma and locally advanced head and neck squamous cell carcinoma (111, 112). AZD3965, a dual MCT1 and MCT2 inhibitor, has previously been shown to be effective in the treatment of breast cancer (113). A recently completed phase 1 clinical trial validated the safety and efficacy of AZD3965 in patients with advanced solid malignancies and lymphoma (114). As a result, we anticipate that investigating Kla will provide more possibilities for tumor treatment.

### 5.2 Lactylation affecting immunotherapy

Kla is crucial in immunotherapy. The therapeutic efficacy of programmed cell death protein 1 (PD-1) blocking therapy is determined by the competition for the reactivation of PD-1-expressing CD8+ T cells and Treg cells in TME (115). Nishikawa et al. (4) showed that Treg cells express more PD-1 than effector T cells in highly glycolytic malignancies. Under low-glucose conditions, Treg cells rapidly absorb lactate through MCT1, stimulating the translocation of the nuclear factor of activated T cell 1 (NFAT1) into the nucleus and consequently increasing PD-1 expression, whereas effector T cells suppress PD-1 expression. Treatment failure has been reported to occur because PD-1 inhibition stimulated PD-1-expressing Treg cells. Furthermore, studies have shown that lactate can also promote the expression of PD-L1 on macrophages and neutrophils to mediate immune resistance (116, 117). Preclinical studies have indicated that combining the MCT1 inhibitor AZD3965 with anti-PD-1 therapy lowers lactate release into the TME and boosts anti-tumor immunity (118). Lu et al. (10) found that Kla levels in the Treg cells of patients with HCC who responded to anti-PD-1 therapy were lower. Moreover, the combination of anti-PD-1 drugs and lactate dehydrogenase inhibitors has a stronger antitumor effect than anti-PD-1 drugs alone. This indicates that combining Kla inhibition with immunotherapy may be effective in treating tumors.

Kla has been found to be abundant on histone and nonhistone proteins in tumor cells, and it is linked to a poor prognosis. Therefore, exploration of Kla and its regulatory sites could help identify additional safe and effective therapeutic targets for cancer treatment and provide new directions for combination therapies.

## 6 Conclusions and future perspectives

Kla plays an important role in tumor metabolic reprogramming, angiogenesis, immune escape, and regulation of intestinal flora. It has also been linked to the occurrence and progression of malignancies. However, Kla-targeting techniques and clinical translation remain in their infancy. Numerous issues have yet to be addressed, such as, the need to investigate the synergistic or antagonistic effects of lactate and other epigenetic modifications to better understand the mechanism of tumor metabolic reprogramming, the need to investigate more nonhistone Kla sites to identify potential “writers” and “erasers,” and the need to investigate the effect of Kla on immune cells for improving the safety and effectiveness of immunotherapy. Targeted suppression of Kla coupled with standard chemotherapy, radiotherapy, and immunotherapy can be expected to provide additional cancer treatment options in the future.

## Author contributions

Conceptualization: YX and XJ. Methodology: JS. Software: ZZ. Validation: YX, and XJ. Formal analysis: SC. Investigation: CB. Resources: XJ. Data curation: ZZ. Writing-original draft preparation: JS and HY. Writing-review and editing: JS and YX. Visualization: XJ. Supervision: XJ. Project administration: XJ. Funding acquisition: XJ. All authors contributed to the article and approved the submitted version.

## Glossary

**Table:**

PTMs	post-translational modifications
Kla	Lactylation
GPR81	Gi-protein-coupled receptor 81
TME	tumor microenvironment
HDAC	histone deacetylase
CBP	CREB-binding protein
HDAC	histone deacetylase
SIRT	sirtuins
La	lactyl;’
NSCLC	non-small cell lung cancer
ATP	adenosine triphosphate
NADH	nicotinamide adenine dinucleotide
TCA	tricarboxylic acid
LDH	lactate dehydrogenase
AMPK	AMP-activated protein kinase
AKT	activating protein kinase B
KLF4	kruppel-like factor 4
NF-κB	nuclear factor-kappa B
MCT1	moncarboxylate transporter 1
TAM	tumor-associated macrophages
VEGF	vascular endothelial-derived growth factor
Arg1	arginase 1
CTLs	cytotoxic T lymphocytes
NK	Natural killer
NKT	Natural killer T
IFNγ	interferon-γ
IL	interleukin
PDK	pyruvate dehydrogenase kinase
DCA	dichloroacetic acid
MGO	methylglyoxal
LGSH	lactoylglutathione
GLO1	glyoxalase 1
ALDOA	aldolase A
AK2	adenylate kinase 2
HCC	hepatocellular carcinoma
HIF-1α	hypoxia-inducible factor alpha
PHD	proline hydroxylase
ENO1	enolase
HK-1	hemokinin-1
PKM	pyruvate kinase M
SDHA	succinate dehydrogenase
IDH3G	isocitrate dehydrogenase 3 gamma
TGF	transforming growth factor
LPS	lipopolysaccharide
PI3K	phosphatidylinositol-3 kinase
TIMs	tumor-infiltrating myeloid cells
METTL3	methyltransferase-like 3
JAK1	janus kinase
PykF	pyruvate kinase I
HP	helicobacter pylori
TLR	toll-like receptors
YY1	yin-yang 1
SDH	succinate dehydrogenase
IDH	lactate dehydrogenase
CCNE2	cyclin E2
PCSK9	proprotein convertase subtilisin-kexin type 9
MIF	macrophage migration inhibitory factor
VHL	von Hippel-Lindau
ccRCC	clear cell renal cell carcinoma
PDGFRβ	platelet-derived growth factor receptor β
YTHDF2	YT521-B homology domain family member 2
PD-1	programmed cell death protein 1
NFAT1	nuclear factor of activated T cells 1
MDSCs	myeloid-derived suppressor cells
STAT3	signal transducer and activator of transcription 3
FoxP3	Forkhead/winged helix transcriptional factor P3
MHC	major histocompatibility complex
LND	lonidamine
